# Prospective Evaluation of Quantitative F-18-FDG-PET/CT for Pre-Operative Thoracic Lymph Node Staging in Patients with Lung Cancer as a Target for Computer-Aided Diagnosis

**DOI:** 10.3390/diagnostics13071263

**Published:** 2023-03-27

**Authors:** Philipp Genseke, Christoph Ferdinand Wielenberg, Jens Schreiber, Eva Luecke, Steffen Frese, Thorsten Walles, Michael Christoph Kreissl

**Affiliations:** 1 Division of Nuclear Medicine, Department for Radiology and Nuclear Medicine, University Hospital Magdeburg, 39120 Magdeburg, Germany; 2 Department of Nuclear Medicine, Faculty of Medicine, Medical Center-University of Freiburg, University of Freiburg, 79106 Freiburg, Germany; 3 Department for Pneumology, University Hospital Magdeburg, 39120 Magdeburg, Germany; 4 Lung Clinic Lostau, 39291 Lostau, Germany; 5 Department of Thoracic Surgery, University Hospital Magdeburg, 39120 Magdeburg, Germany; 6 Research Campus STIMULATE, Otto-Von-Guericke University, 39106 Magdeburg, Germany

**Keywords:** PET/CT, lung cancer, lymph node metastases, quantification, computer aided diagnosis

## Abstract

Purpose: Pre-operative assessment of thoracic lymphonodal (LN) involvement in patients with lung cancer (LC) is crucial when choosing the treatment modality. Visual assessment of F-18-FDG-PET/CT (PET/CT) is well established, however, there is still a need for prospective quantitative data to differentiate benign from malignant lesions which would simplify staging and guide the further implementation of computer-aided diagnosis (CAD). Methods: In this prospective study, 37 patients with confirmed lung cancer (m/f = 24/13; age: 70 [52–83] years) were analyzed. All patients underwent PET/CT and quantitative data (standardized uptake values) were obtained. Histological results were available for 101 thoracic lymph nodes. Quantitative data were matched to determine cut-off values for delineation between benign vs. malignant lymph nodes. Furthermore, a scoring system derived from these cut-off values was established. Statistical analyses were performed through ROC analysis. Results: Quantitative analysis revealed the optimal cut-off values (*p* < 0.01) for the differentiation between benign and malignant thoracic lymph nodes in patients suffering from lung cancer. The respective areas under the curve (AUC) ranged from 0.86 to 0.94. The highest AUC for a ratio of lymph node to healthy lung tissue was 0.94. The resulting accuracy ranged from 78.2% to 89.1%. A dedicated scoring system led to an AUC of 0.93 with a negative predictive value of 95.4%. Conclusion: Quantitative analysis of F-18-FDG-PET/CT data provides reliable results for delineation between benign and malignant thoracic lymph nodes. Thus, quantitative parameters can improve diagnostic accuracy and reliability and can also facilitate the handling of the steadily increasing number of clinical examinations.

## 1. Introduction

In 2014, the European Society of Thoracic Surgeons (ESTS) updated the algorithm for preoperative mediastinal lymph node staging for non-small cell lung cancer (NSCLC). In this algorithm, F-18-fluorodesoxyglucose (FDG) positron emission tomography/computer tomography (PET/CT) imaging plays a key role in the characterization of hilar and mediastinal lymph nodes [1,2,3]. Nevertheless, a meta-analysis by Birim and colleagues demonstrated that the overall sensitivity for the detection of lymph node metastases in PET was about 83% and the overall specificity was 92% [4]. Therefore, invasive methods such as transbronchial biopsy and mediastinoscopy are still the gold standard for the final exclusion of lymph node metastases, because their specificity is, by definition, 100% [5,6].

At present, there are several diagnostic criteria, occasionally with significant discrepancies, to classify a lymph node in PET/CT as benign or malignant. The criterion of a maximum standard uptake value (SUVmax) ≥ 2.5 achieved an overall sensitivity of 81.3% and an overall specificity of 79.4%, as reported by Schmidt–Hansen and colleagues [7]. Another approach to classify a lymph node in PET/CT is the calculation of SUV ratios to healthy tissues (e.g., liver) or the primary tumor. Mattes et al. were able to increase the accuracy in prediction of nodal malignancy in certain lymph nodes by using a ratio of lymph node to primary tumor [8]. With a ratio of lymph node to liver, Nguyen et al. correctly classified 88.3% of the analyzed lymph nodes and achieved a sensitivity of 83.9% with a specificity of 97.6% [9].

Analogous to the Deauville Score, for example, there are similar approaches for visually comparing lymph nodes with other organs in patients with lung cancer [10]. With a visual reading score, our institution achieved accuracies of 91.4% to 93.5% and negative predictive values of 97.3–97.6%, as recently published [11,12].

Nevertheless, none of these parameters or scores have been implemented in clinical routines so far, most likely due to missing reproducibility or unsatisfactory performance as well as a lack of prospective data. Therefore, standardized, reproducible, and especially prospective evaluated criteria are needed to optimize diagnostics and clinical care. Due to the fast development of artificial-intelligence-based-quantitative-analysis and its expected role in future medicine, these criteria preferably should be of a quantitative nature as they represent a promising target for computer-aided diagnosis [9,13,14].

The aim of this prospective study was therefore to find reliable and reproducible quantitative diagnostic parameters which (a) have a high diagnostic accuracy and (b) can be easily obtained from PET/CT in clinical routine.

## 2. Materials and Methods

### 2.1. Patients

This monocentric prospective study was approved by the local ethics committee (registration number 115/17) and registered at the WHO-clinical-trial database (ID: DRKS00012624).

We prospectively recruited 73 (female, *n* = 22; male, *n* = 51) patients in our department from August 2017 to February 2018. Due to the lack of at least one criterion (see below) in the further course, 36 patients were excluded from evaluation (dropout rate: 49.3%).

37 patients (female, *n* = 13; male, *n* = 24; median age 70 years [range, 52–83 years]) with a total of 101 assessable lymph nodes (mean diameter, 13.7 ± 10 mm) and a PET/CT during the course of clinical routine were prospectively included.

All analyzed lymph nodes were confirmed histologically (preferably through surgery or mediastinoscopyor transbronchial needle biopsy), which served as the standard of reference.

Patients meeting the following criteria were included in the study: (a) histologically confirmed lung cancer (primary tumor), (b) histologically confirmed or excluded lymph node involvement, (c) fasting blood glucose level ≤ 8.3 mmol/L on the day of the PET/CT, and (d) uptake time between 45–70 min after injection.

Histological subtypes included small cell lung cancer (*n* = 1), adenocarcinoma (AC, *n* = 19), squamous cell carcinoma (SCC, *n* = 16), and non-small cell lung cancer not otherwise specified (NSCLC NOS, *n* = 1).

### 2.2. PET/CT Imaging

PET/CT imaging was performed using an EARL-certified PET/CT device (Biograph mCT 64^®^; Siemens Health-care, Erlangen, Germany). Imaging was performed in accordance with the current procedural guidelines of the European Association of Nuclear Medicine [15]. A median activity of 236 MBq F-18-FDG (IQR, 232–239 MBq; range, 226–245 MBq) was intravenously administered and the PET scan started after a median uptake time of 56 min (IQR, 55–58 min; range, 46–68 min). PET data were acquired from the base of the skull to mid-thigh (Six to eight bed position; three minutes each and axial coverage of 216 mm per bed position and an overlap of 89 mm). The low-dose CT was used for attenuation correction and anatomical mapping (tube current, 50 mA; tube voltage, 120 kV; gantry rotation time 0.5 s; pitch 0.8).

### 2.3. PET/CT Image Reconstruction

PET raw data were reconstructed using iterative reconstruction with system-specific point spread function (PSF) modelling and time of flight (TOF) analysis (Siemens TrueX^®^, UltraHD PET^®^; iterations: 2; subsets: 21). Projection data were reconstructed with a 5 mm slice thickness (rows: 512; columns: 512; voxel size: 1.5 × 1.5 × 5.0 mm). After reconstruction, a Gaussian filter (full width half maximum [FWHM], 2 mm) was applied. CT raw data were reconstructed with a slice thickness of 5 mm and a special filter for low-dose CT (convolution kernel, B19f).

### 2.4. Quantification

For quantitative image analysis of the corrected PET datasets, we used the software ROVER^®^ (Version: 2.1.40, ABX advanced biochemical compounds GmbH; Radeberg; Germany).

Volumes of interest were manually assigned to the lymph node to be analyzed and the respective quantitative parameters were measured. Liver standard uptake value was calculated by placing a volume of interest with a diameter of 5 cm in the right lobe of the liver. Standard uptake value of the brainstem was assessed with a 2 cm volume of interest in the co-imaged brainstem. Standard uptake value in healthy lung tissue was evaluated by placing a volume of interest with a diameter of 5 cm in the lung contralateral to the tumor. Standard uptake value of the primary was calculated by placing an appropriate volume of interest around the primary. We then compiled the quotients, which are detailed in Table 1.

Additionally, we also addressed recently published data on visual reading [11,12]. In those publications, a visual reading score was utilized. The scoring system was defined as follows: uptake of lymph node appears ≤ mediastinal blood pool structures (score 1); uptake of lymph node appears > uptake of mediastinal blood pool structures but < uptake of the liver (score 2); uptake of lymph node appears ≥ uptake of the liver but not black (score 3); uptake of lymph node appears ‘black’ (score 4). With a cut-off score higher than three, a sensitivity between 88.9% and 90.7% with a specificity between 92.0% and 94.6% was achieved [11]. Addressing this publication, we now quantified this score as follows: maximum uptake of lymph node ≤ maximum uptake of liver (score 1); maximum uptake of liver < maximum uptake of lymph node ≤ maximum uptake of primary (score 2); maximum uptake of primary < maximum uptake of lymph node (score 3).

Furthermore, we assessed a multifactorial scoring system, which consisted of these five conditions: (I) SUVmax ≥ 5.495, (II) ratio lymph node to primary ≥ 0.457, (III) ratio lymph node to liver ≥ 1.374, (IV) ratio lymph node to brainstem ≥ 0.749, and (V) ratio lymph node to healthy lung tissue ≥ 4.593. For each fulfilled condition, a point was obtained.

### 2.5. Statistical Analyses

Data analysis was performed with IBM SPSS Statistics 24 (IBM Corporation, Armonk, NY, USA).

Descriptive parameters were expressed as median, IQR, and range, unless otherwise specified. Furthermore, we calculated receiver operating characteristic (ROC) curves and the corresponding areas under the curve (AUC) with their 95% confidence interval (95%-CI).

The optimal cut-off values were defined as the points with the smallest distance to the point (0· 1) on the ROC curve and were calculated with the following formula:

(1) $$d=\sqrt{{\left(1-Sensitivity\right)}^{2}+{\left(1-Specifity\right)}^{2}}$$

The resulting diagnostic performance expressed by sensitivity, specificity, positive predictive value (PPV), negative predictive value (NPV), and accuracy at computed cut-off values was evaluated using standard formulas.

Significance was assumed at *p*-values < 0.05.

## 3. Results

Overall, 101 lymph nodes could be identified in PET and low-dose CT data (median lymph nodes per patient, *n* = 3; range, 1–6). The lymph nodes were classified into the staging system according to Mountain and Dresler [16]. The distribution of lymph nodes was as follows: EBUS 2 (*n* = 6), 4 (*n* = 21), 5 (*n* = 8), 6 (*n* = 3), 7 (*n* = 27), 10 (*n* = 18), 11 (*n* = 16), and 12 (*n* = 2). Twenty-nine of those lymph nodes (28.7%) were malignant and seventy-two of those lymph nodes (71.3%) had a benign histology.

### 3.1. SUV Measurements

Significant differences between benign and malign lymph nodes were seen for SUVmax, SUVmean, SUVpeak, ratio SUVmax lymph node to SUVmax primary, ratio SUVmax lymph node to SUVmax liver, ratio SUVmax lymph node to SUVmax brainstem, and ratio SUVmax lymph node to SUVmax of contralateral lung, with *p* < 0.05, respectively. Detailed results are shown in Table 2.

### 3.2. ROC Analyses and Diagnostic Performance

A SUVmax of 5.5 as the optimum cut-off value led to the highest overall accuracy (89.1%) with a resulting AUC of 0.92 (Confidence interval (CI): 0.85–0.98). Sensitivity, specificity, negative predictive value, and positive predictive value for SUVmax were 89.67%, 88.89%, 95.5%, and 76.5%, respectively.

The highest negative predictive value with 100% was achieved for ratio lymph node to healthy lung tissue, with an AUC of 0.94 (CI: 0.89–0.98). Sensitivity, specificity, positive predictive value, and accuracy were 100%, 73.6%, 60.4%, and 81.9%.

Ratio lymph node to brainstem led to the lowest observed accuracy (79.21%) with a resulting AUC of 0.88 (CI: 0.81–0.95). Sensitivity, specificity, negative predictive value, and positive predictive value were 82.7%, 77.8%, 91.8%, and 60.0%.

The lowest observed AUC was 0.86 (CI: 0.78–0.94) for ratio lymph node to primary tumor, resulting in an overall accuracy of 80.2%. Sensitivity, specificity, negative predictive value, and positive predictive value were 79.3%, 80.6%, 90.6%, and 62.2%.

Ratio of lymph node to liver led to an accuracy of 84.2% with an area under the curve of 0.91 (CI: 0.85–0.98). Sensitivity, specificity, negative predictive value, and positive predictive value were 89.6%, 81.9%, 95.2%, and 66.7%.

The areas under the curves with their respective cut-off values are shown in Table 3; for cross tables, see Table 4.

### 3.3. Scoring System Adapted from Visual Reading Score

Addressing our recent publication with a visual reading score to predict malignancy in thoracic lymph nodes, we now quantified this score as aforementioned [11,12]. The best diagnostic performance was achieved for a cut-off score higher than two (maximum uptake of primary < maximum uptake of lymph node) with an AUC of 0.81 (CI: 0.72–0.89). This led to an accuracy of 78.2%. Sensitivity, specificity, negative predictive value, and positive predictive value were 34.5%, 95.9%, 78.4%, and 76.9%.

### 3.4. Dedicated Scoring System

Furthermore, we assessed a multifactorial scoring system, outlined above. The ROC analyses for this score revealed an optimal cut-off point for a score higher than two with an AUC of 0.93 (CI: 0.88–0.98) and an accuracy of 87.1%. Sensitivity, specificity, negative predictive value, and positive predictive value were 89.7%, 86.1%, 95.4%, and 72.2%.

### 3.5. Patient Examples

In some cases, the quantitative values did not match the histological results.

In an 80-year-old patient, a perifocal inflammatory reaction was confirmed in the pathological report of the lymph node, which was falsely classified in PET as being positive. This inflammation led to an increase of the SUVmax of the lymph node of 10.1 and was thus difficult to distinguish from a malignant lymph node.

In another case, PET/CT classified a lymph node with a SUVmax of 5.7 as malignant. Nevertheless, histology outruled metastasis but showed a mixed dust pneumoconiosis. Additionally, the consideration to balance those inflammatory-related uptake patterns by using a ratio of lymph node to non-tumor-affected lung tissue did not work. With a value of 7.0, this lymph node was evaluated as malignant.

A false negative lymph node (SUVmax: 3.45) was seen in a 77-year-old female patient. Here, due to the presentation in CT images and the uptake pattern in PET, the presence of a partly lepidic adenocarcinoma can be assumed. According to Suarez–Piñera and colleagues, these led to a lower standard uptake value compared with other lung cancer entities [17].

## 4. Discussion

In this prospective study, we investigated whether there are quantifiable parameters in FDG-PET/CT that distinguish between malignant and benign lymph nodes in patients with lung cancer. The definition of these parameters are essential for the implementation of computer-aided diagnosis and potentially machine learning, which are on the rise in the field of medical applications. As of today, research and applications are mainly focusing on the field of neurological research [18,19]. Nevertheless, this technology is also finding its way into other research areas of medicine and medical imaging [20,21].

Individualized therapy is further gaining importance in lung cancer treatment, but the necessary diagnostic procedures are becoming more complex and elaborate. Machine learning and computer-aided diagnosis could help to save resources, especially physicians’ time in clinical practice. Furthermore, in this way, interrater differences common in the visual interpretation of PET may be reduced. The results of imaging would show a better reproducibility, which may facilitate its use in multicenter studies.

**Figure 1:**
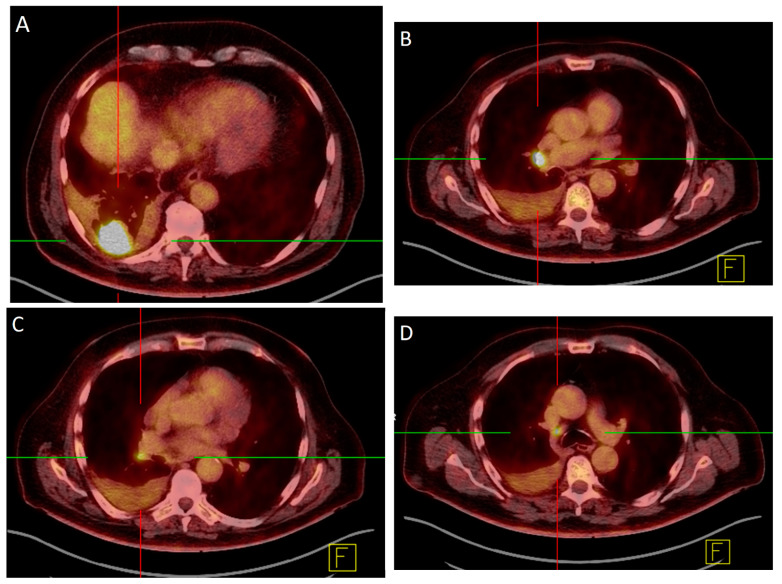
78-year-old male with a histologically proven adeno carcinoma in the right lower lobe (**A**). The left hilar region showed involvement in the EBUS region 10R (**B**) but not 11R (**C**). An additional lymph node in the region 4R (**D**) was positive as well. All findings were confirmed histologically.

In this study, we investigated quantitative parameters that may be used further in computer-aided diagnosis and machine learning. A clinical example is given in Figure 1. A SUVmax threshold is a commonly used parameter, which was also examined in this study [7,13]. On the basis of this threshold, an area under the curve of 0.92 (CI: 0.85–0.98), a sensitivity of 89.7%, and a specificity of 88.9% were reached. Therefore, it allows for an almost perfect differentiation between benign and malignant lymph nodes. In particular, the very high negative predictive value might help reduce further diagnostic procedures in PET-negative lymph nodes. Our data is consistent with the results of Perigaud and colleagues, who concluded from their study that patients with PET-negative lymph nodes can be referred resection of the primary tumor without invasive mediastinal staging [14].

Another quantitative parameter that we investigated was the ratio lymph node to primary tumor, which has also been investigated in some studies [8,9,22]. A ratio to the primary tumor can possibly equalize differences in uptake due to different histological subtypes [23]. In our study, we found an area under the curve of 0.86 (CI: 0.78–0.94) for the ratio of lymph node to primary with a sensitivity of 79.3% and a specificity of 80.6%, which is higher compared with other authors such as Nguyen and colleagues who achieved a sensitivity of 73.0% and a specificity of 77.1%, also resulting in a higher accuracy [9].

A possible explanation for this finding is the very strict institutional PET/CT protocol in our study, including a very strict limit in uptake time (median uptake time: 56 min; range: 46–68 min).

The ratio of lymph node to liver has not been frequently studied but is nevertheless of interest [9,24]. As hepatic FDG uptake correlates closely with blood glucose levels, the ratio of lymph node to liver uptake could be used for the correction of interindividual differences in glucose metabolism [25,26]. For this approach, we were able to show an area under the curve of 0.91 (CI: 0.85–0.98), a sensitivity of 89.7%, and a specificity of 81.9% which is in line with Tournoy and colleagues who showed similar performances using a slightly different patient preparation (e.g., use of diazepam) and the SUVmean of the liver instead of the SUVmax [24]. Nevertheless, our results are not superior to simply using a SUVmax threshold. Maybe the approach to correct interindividual differences in glucose metabolism had a minor impact, due to the strict blood glucose limit (blood glucose ≤ 8.3 mmol/L before injection) in our institution.

Moreover, we assessed two ratios to the brainstem and non-affected contralateral lung tissue that were not found in the literature so far.

The ratio of lymph node to brainstem was calculated because we assumed that the brainstem has a fairly constant FDG uptake rate and the SUV of this region more accurately reflects the availability of FDG [27]. The respective ratio has an area under the curve of 0.88 (CI: 0.81–0.95) with a sensitivity of 82.8% and a specificity of 77.8%. With an accuracy of 79.2%, this was the least accurate ratio that we found. This is contradictory to preclinical tumor to brain evaluations on tumor-bearing mice [28].

However, preclinical data cannot be readily translated to humans. Vigliant and colleagues found a strong dependency of brain SUV on blood glucose levels [29]. Another study by Britz–Cunningham and colleagues found a superiority of uptake ratio with the brain (cortex, basal ganglia, or cerebellum) as a reference [30]. In our study, we used the brainstem because it was still located within the field of view (thighs to base of skull). Upon visual evaluation, the area was free of artefacts in spite of being located at the end of the field of view, however, quantitative parameters could still have been influenced, resulting in our observation.

In addition, we were able to achieve a good performance with the ratio of lymph node to the contralateral, non-tumor-affected lung tissue. Patients with lung cancer often suffer from other lung diseases (e.g., chronic obstructive pulmonary disease), which affects glucose metabolism in the mediastinal and hilar lymph nodes. Using a ratio to the contralateral non-tumor-affected lung tissue could balance inflammatory-related changes in FDG uptake, because FDG uptake of lung tissue in patients with chronic obstructive pulmonary disease (COPD) is higher compared with patients with healthy lung tissue [31]. The ratio of lymph node to healthy lung tissue achieved an area under the curve of 0.94 (CI: 0.89–0.98) with a sensitivity of 100%, an accuracy of 81.9%, and a negative predictive value of 100%. Even if the assumed effect of increasing specificity does not occur, high sensitivity with acceptable specificity is very interesting for computer-aided diagnosis.

Scoring systems play a role in the diagnosis of various diseases, for example, the TIRADS classification for the management of thyroid nodules [32]. The advantage of those scoring systems is the integration of several characteristics. With an accuracy of 87.1%, this score was very accurate in predicting nodal malignancy but had no advantage compared with SUVmax or other parameters. Even though this approach had no benefit in this study, further investigation could be valuable. Since CT parameters are automatically collected in addition to PET parameters, it may be useful to include them in a further score. For example, lymph node size or, even better, the change in lymph node size compared with previous examinations could be included.

As demonstrated earlier, a strictly visual reading is suitable in clinical routine, delivering high accuracy rates [12]. Nevertheless, visual impressions are not numerical data and therefore, implementation of progressive techniques such as computer-aided diagnosis systems is impeded. Consequently, we tried to ‘translate’ visual reading into quantitation as described. Unfortunately, the performance of this approach was not nearly as good as in our initial study and was inferior to the aforementioned values and ratios. This might be due to the fact that quantitation is intrinsically more precise than subjective image impression. In addition, the ‘translation’ of the condition ‘uptake of lymph node appears black’ was difficult and led to the condition uptake of primary < uptake of lymph node, which is obviously inaccurate. This does not mean that visual reading is the wrong approach in clinical routine at present; the data suggests that the thresholds we used initially are not suitable for machine learning.

A potential limitation of this work is the considerably small number of patients (*n* = 37) and lymph nodes examined (*n* = 101). Due to the monocentric approach with only one PET/CT device, a direct application of the results to other institutions might only be partially possible. The presence of the different histological subtypes of lung carcinoma can limit the use of quantitative PET parameters, as demonstrated in one example. In further studies with larger numbers of patients, a differentiation with regard to different subtypes would be an interesting approach.

## 5. Conclusions

Lymph node SUVmax and ratio lymph node to lung tissue, derived from a dedicated F-18-FDG-PET/CT, allow for a reliable differentiation between benign and malignant thoracic lymph nodes in patients with lung cancer.

In the face of increasing examination numbers in PET/CT, those data could represent a promising target for an effective and efficient diagnostic approach. This could not only help physicians in clinical routine but might also lead to a more precise and reproducible practice in clinical care.

## Figures and Tables

**Table 1:** Calculation of the named ratios.

Lymph node/primary	SUVmax lymph node/SUVmax primary
Lymph node/liver	SUVmax lymph node/SUVmax liver
Lymph node/brainstem	SUVmax lymph node/SUVmax brainstem
Lymph node/lung	SUVmax lymph node/SUVmax healthy lung tissue

**Table 2:** Quantitative parameters measured in PET/CT; values shown are median and range.

	Benign	Malign
**SUVmax**	3.45 (1.14–19.00)	11.00 (2.35–35.55)
	*p* < 0.05	
**Lymph node/primary**	0.22 (0.06–1.27)	0.75 (0.13–2.42)
	*p* < 0.05	
**Lymph node/liver**	0.92 (0.28–4.42)	3.18 (0.63–11.19)
	*p* < 0.05	
**Lymph node/brainstem**	0.52 (0.14–3.88)	1.63 (0.51–8.46)
	*p* < 0.05	
**Lymph node/lung**	3.47 (1.08–19.00)	14.67 (4.60–50.61)
	*p* < 0.05	

**Table 3:** ROC analyses and diagnostic performance of the above ratios and scores.

	Cut-Off (*p* < 0.001)	AUC	Sens	Spez	PPV	NPV	Acc
**SUVmax**	5.495	0.92 (CI: 0.85–0.98)	89.67%	88.89%	76.47%	95.52%	89.11%
**Lymph node/primary**	0.457	0.86 (CI: 0.78–0.94)	79.31%	80.56%	62.16%	90.63%	80.20%
**Lymph node/liver**	1.374	0.91 (CI: 0.85–0.98)	89.66%	81.94%	66.67%	95.16%	84.16%
**Lymph node/brainstem**	0.749	0.88 (CI: 0.81–0.95)	82.76%	77.78%	60.00%	91.80%	79.21%
**Lymph node/healthy lung**	4.593	0.94 (CI: 0.89–0.98)	100%	73.61%	60.42%	100%	81.89%
**Adapted visual score**	≥3	0.81 (CI: 0.72–0.89)	34.48%	95.83%	76.92%	78.41%	78.22%
**Score**	≥3	0.93 (CI: 0.88–0.98)	89.66%	86.11%	72.22%	95.38%	87.13%

**Table 4:** Cross tables for the respective ratios.

	··	PET Neg	PET Pos
**SUVmax;** **cut-off 5.495**	**Histo neg**	64	8
	**Histo pos**	3	26
**Lymph node/primary;** **cut-off 0.457**	··	**PET neg**	**PET pos**
	**Histo neg**	58	14
	**Histo pos**	6	23
**Lymph node/liver;** **cut-off 1.374**	··	**PET neg**	**PET pos**
	**Histo neg**	59	13
	**Histo pos**	3	26
**Lymph node/brainstem;** **cut-off 0.749**	··	**PET neg**	**PET pos**
	**Histo neg**	56	16
	**Histo pos**	5	24
**Lymph node/lung;** **cut-off 4.593**	··	**PET neg**	**PET pos**
	**Histo neg**	53	19
	**Histo pos**	0	29

## Data Availability

The data presented in this study are available on request from the corresponding author. The data are not publicly available due to privacy restrictions.
